# Subarachnoid small vein occlusion due to inflammatory fibrosis—a possible mechanism for cerebellar infarction in cryptococcal meningoencephalitis: a case report

**DOI:** 10.1186/s12883-017-0934-y

**Published:** 2017-08-09

**Authors:** Yoshiteru Shimoda, Satoru Ohtomo, Hiroaki Arai, Takashi Ohtoh, Teiji Tominaga

**Affiliations:** 1 Department of Neurosurgery, South Miyagi Medical Center, 38-1 Azanishi, Ogawara-machi, Shibata-gun, Miyagi 989-1253 Japan; 2 Department of Neurosurgery, Tohoku University Graduate School of Medicine, Sendai, Japan; 3 Department of Pathology, South Miyagi Medical Center, Shibata-gun, Miyagi Japan

**Keywords:** Cryptococcal infection, Vein occlusion, Magnetic resonance imaging, Autopsy

## Abstract

**Background:**

Cryptococcal meningoencephalitis (CM) causes cerebral infarction, typically, lacunar infarction in the basal ganglia. However, massive cerebral infarction leading to death is rare and its pathophysiology is unclear. We report a case of CM causing massive cerebellar infarction, which led to cerebral herniation and death.

**Case presentation:**

A 56-year-old man who suffered from dizziness and gait disturbance for one month was admitted to our hospital and subsequently diagnosed with a cerebellar infarction. He had a past medical history of hepatitis type B virus infection and hepatic failure. Although the findings on magnetic resonance imaging (MRI) imitated an arterial infarction of the posterior inferior cerebellar artery, an accompanying irregular peripheral edema was observed. The ischemic lesion progressed, subsequently exerting a mass effect and leading to impaired consciousness. External and internal decompression surgeries were performed. *Cryptococcus neoformans* was confirmed in the surgical specimen, and the patient was diagnosed with CM. In addition, venule congestion in the parenchyma was observed with extensive fibrosis and compressed veins in the subarachnoid space. The patient died 26 days after admission. Autopsy revealed that pathological changes were localized in the cerebellum.

**Conclusion:**

*C. neoformans* can induce extensive fibrosis of the subarachnoid space, which may compress small veins mechanically inducing venule congestion and massive cerebral infarction. In such cases, the clinical course can be severe and even rapidly fatal. An atypical pattern of infarction on MRI should alert clinicians to the possibility of *C. neoformans* infection.

## Background

Cryptococcal meningoencephalitis (CM) caused by *Cryptococcus neoformans* infection is the most common fungal infection of the central nervous system [1, 2]. Throughout its clinical course, *C. neoformans* induces small cerebral infarctions such as lacunar infarctions primarily in the basal ganglia [3–8]; however, massive brain infarction causing cerebral herniation has not been well described. We present a case of a patient with CM who presented with an atypical cerebellar infarction, which ultimately caused fatal cerebral herniation. Pathological and radiological findings revealed possible underlying pathogenesis.

## Case presentation

**Fig. 1:**
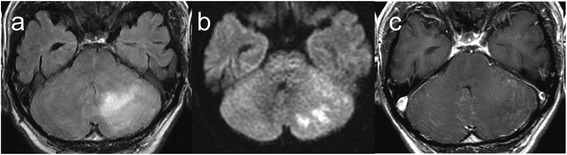
Brain magnetic resonance imaging performed on admission. The findings on diffusion weighted imaging (DWI) mimicked arterial infarction of the posterior inferior cerebellar artery accompanying strong perilesional edema with almost no enhancement of the lesion. **a** Fluid-attenuated inversion recovery (FLAIR) image on admission. **b** DWI. **c** T1-weighted imaging with gadolinium enhancement

**Fig. 2:**
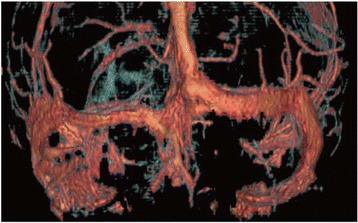
Reconstruction of gadolinium enhanced magnetic resonance imaging performed on admission. Although sinus venous thrombosis was suspected to be the cause of the observed cerebellar infarction with edema, the main venous sinuses were confirmed to be patent

A 56-year-old man presented to our department with headache, vomiting, and gait disturbance (for 1 month). He had a past medical history of hepatitis type B virus infection and hepatic failure. He had been medically treated for hypertension and hepatitis for the previous 4 years. On admission, he had an impaired consciousness [Glasgow coma scale (GCS), 14]. Cerebellar ataxia and gait disturbance were evident. Diffusion weighted imaging (DWI) demonstrated multiple cerebellar infarctions at several intensities with perilesional edema of the left cerebellar hemisphere (Fig. 1a, b). Brain magnetic resonance imaging (MRI) did not reveal any prominent meningeal gadolinium enhancement or nodule (Fig. 1c). MR angiography revealed no abnormal findings. The main venous sinuses were confirmed to be patent by 3-dimensional reconstructions of MRI with gadolinium (Fig. 2). Chest X-ray did not reveal any abnormal lesions, and the results of serum examination for infectious diseases, including human immunodeficiency virus (HIV), were negative except for hepatitis B virus surface antigen.

**Fig. 3:**
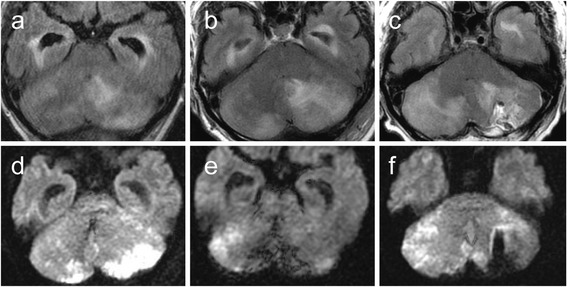
Progression of cerebellar infarction in Cryptococcal meningoencephalitis. **a**, **d** FLAIR (**a**) and DWI (**d**) performed following internal and external decompression 10 days after admission. The ischemic lesion progressed to the contralateral cerebellar hemisphere with peripheral edema and ventricular dilatation unresponsive to external continuous ventricular drainage. **b**, **e**. FLAIR (**b**) and DWI (**e**) performed 17 days after admission. The ischemic and edematous lesion progressed from dorsal to ventral. **c**, **f** FLAIR (**c**) and DWI (**f**) performed 23 days after admission, 3 days before the patient died. The cerebellum had completely swollen, and tight cisterns were observed indicating brain herniation

Subsequently, the patient was diagnosed with subacute cerebellar infarction due to arteriosclerosis and was administered glycerol to control the intracranial pressure; however, 1 week after admission, his GCS decreased to 11. Computed tomography confirmed worsened cerebellar edema and hydrocephalus. External and internal decompression surgery were performed to control the intracranial pressure (Fig. 3a, d). A section of the swollen cerebellar hemisphere was removed and submitted as a surgical specimen. Additionally, external continuous ventricular drainage was performed to control hydrocephalus. Lumbar puncture to collect cerebral spinal fluid (CSF) was not performed until this time because of the risk of cerebral herniation. CSF from continuous ventricular drainage demonstrated mild inflammation (cell count, 36 /mm^3^; protein, 16 mg/dl; glucose, 113 mg/dl). *C. neoformans* was detected in CSF as well as in the surgical specimen of the cerebellum.

**Fig. 4:**
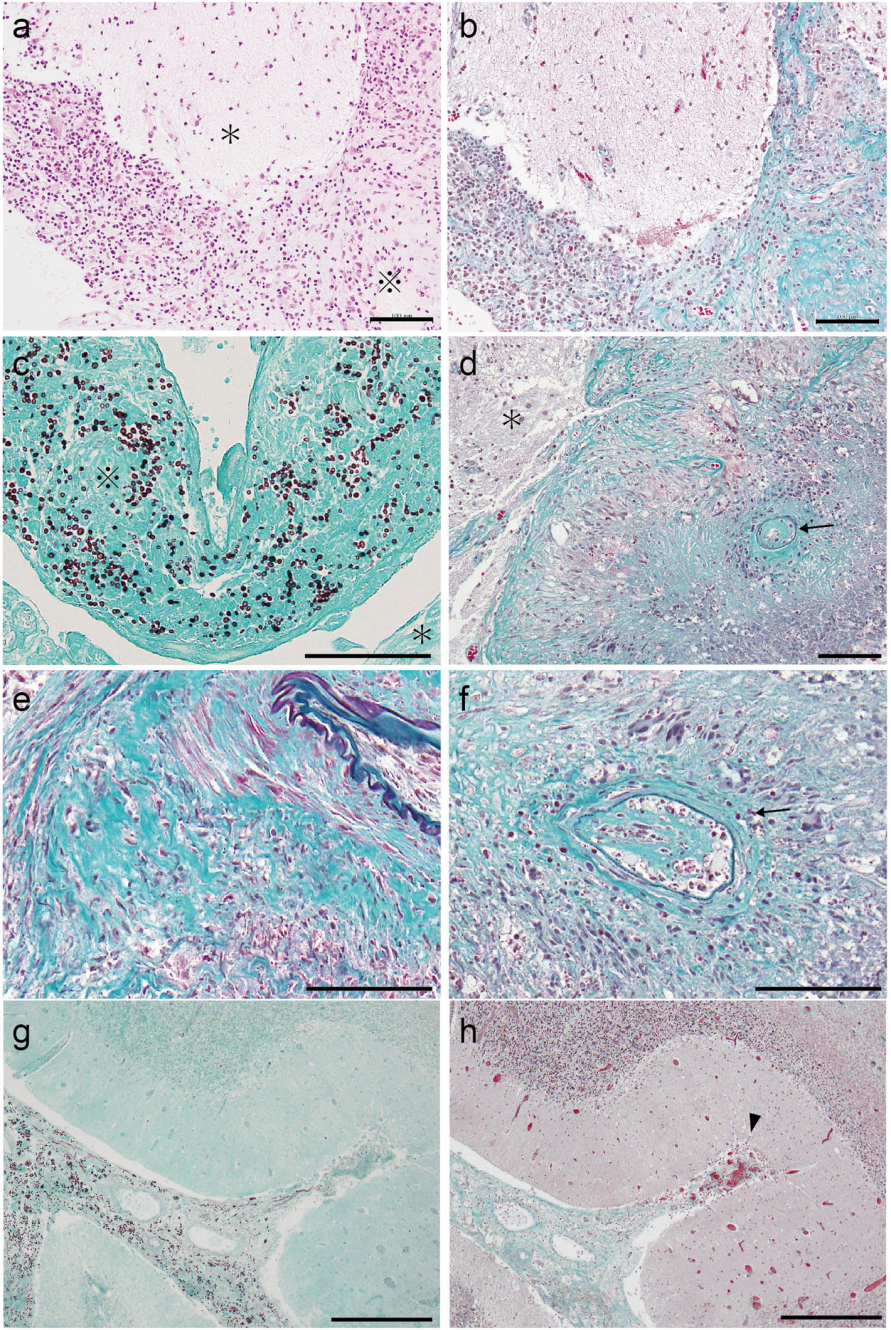
Pathological findings of the cerebellum specimen. **a** Hematoxylin and Eosin staining of the left cerebellar hemisphere from the surgical specimen. The subarachnoid space was heavily thickened with inflammatory cells, which is expressed as ※. The parenchyma is expressed as *. Scale bar: 100 μm. **b** Elastica–Masson staining at the same location as in (**a**). Fibrosis was widely observed in the subarachnoid space, which appears green. Scale bar: 100 μm. **c** Grocott staining of left cerebellar hemisphere from the surgical specimen. Fungi were observed in the subarachnoid space (※) and few fungi were observed inside the parenchyma (*). Scale bar: 100 μm. **d**, **e**, **f** Elastica–Masson staining of the left cerebellar hemisphere from the surgical specimen. Strong and diffuse fibrosis was observed in the subarachnoid space (**e**). Proliferation of endothelial cells was observed inside the inner cavity of small arteries (*arrows* in **d**, **f**), which implies the slow progression of arterial occlusion. Veins were rarely observed in the subarachnoid space, which indicates that they were compressed and occluded. The parenchyma is expressed as *. Scale bar: 100 μm. **g** Grocott staining of the deep sulcus in the left cerebellar hemisphere from the autopsy specimen. The subarachnoid space was filled with fungi not only at the surface of the cerebellar hemisphere but also in the deep sulcus. Scale bar: 500 μm. **h** Elastica–Masson staining at the same location as in (**e**). Fibrosis and thickening of the subarachnoid space was observed even in the deep sulcus of the cerebellar hemisphere. Venules from the parenchyma were frequently observed to be congested at the entry to the fibrinous subarachnoid space (*arrow head*). Scale bar: 500 μm

Histopathologic examination of the surgical specimen revealed strong hyperplasia of the arachnoid mater (Fig. 4a). Fungi were mainly localized in the subarachnoid space and rarely in the parenchyma (Fig. 4c). Lymphocytes and multinucleated giant cells forming granulomata invaded the arachnoid and subarachnoid spaces and pia with heavy fibrosis (Fig. 4b–f). Small arteries were occasionally observed to be occluded with internal endothelial proliferation. While there were arteries in the sample, veins were rarely observed in the subarachnoid space (Fig. 4d). In addition, venules in the parenchyma were frequently observed to be congested. The patient was diagnosed with granulomatous meningitis due to *C. neoformans* and was immediately treated using liposomal amphotericin B and fluconazole; however, the ischemic lesion of the cerebellum continued to bilaterally worsen along with worsening perilesional edema (Fig. 3b–f). The patient’s course subsequently deteriorated. He developed renal failure and ultimately died 25 days after admission.

Autopsy confirmed that the pathological changes were confined to the central nervous system and predominantly localized at the surface of the cerebellar hemisphere. Fungal bodies were widely spread along the surface and bilaterally into the deep sulcus of the cerebellum (Fig. 4g). Few fungi were observed in the supratentorial and intraparenchymal lesions. The lesion in the arachnoid mater of the cerebellum was roughly the same as that in the surgical specimen although it was more deeply spread into the peripheral sulcus and the granulomatous inflammation was not as severe (Fig. 4h).

## Discussion

We observed two key results in this study: CM can result in massive infarction by subarachnoid small vein occlusion and an atypical infarction on MRI can be observed.

Firstly, *C. neoformans* can induce extensive subarachnoid small vein occlusion due to inflammatory fibrosis leading to poor outcomes. Generally, *C. neoformans* tends to hematogenously spread to the brain along the surface, thus inducing meningitis. When the fungi reach the perivascular space of perforating arteries, they begin to invade the perivascular space toward the deeper parts of the brain, simultaneously presenting cerebral infarction mimicking lacunar infarction; however, the mechanism of this process has not been described previously [4, 5]. In contrast, the most unprecedented finding of our case was that the fungi were present not only at the superficial subarachnoid space of the cerebellum but also diffusely spread and deeply within the sulcus (Fig. 4b–h). Peripheral subarachnoid spaces were thickened with increased internal elastic fibers (Fig. 4b–h). While small arteries showed proliferation of endothelial cells (Fig. 4d, f arrows), veins were rarely observed, which indicates that they had been compressed. Meanwhile, venules from the parenchyma were frequently observed to be congested at the entry of the fibroid subarachnoid space (Fig. 4h, arrow head). It can be speculated that the wide spread fungal involvement triggered granulomatous inflammation, which subsequently induced fibrosis. The compression of small veins in the subarachnoid space may have been due to thickened fibroid tissue, thus blocking the venous return from the parenchyma. This may consequently lead to venous infarction with accompanying massive edema and mass effect. Although the broadly scattered fungi may underlie this pathophysiology, it was unclear how the fungi could widely invade the subarachnoid space and deeply invade the cerebellum.

Secondly, the temporal change of the infarction on MRI was unusual compared with that in the previous cases of CM. Cerebral infarctions are observed in 4–32% of patients with CM on MRI, typically lacunar at the basal ganglia [6–9]. In our case, multiple infarctions with variable phases in the left cerebellar hemisphere mimicked arterial infarctions of the posterior inferior cerebellar artery with edema on admission MRI (Fig. 1a, b). Subsequently, the ischemic lesion progressed independent of the territory of the artery (Fig. 3b–f). The lesion gradually spread from the left to the right cerebellar hemisphere and from the surface to deeper parts of the cerebellum (Fig. 3b–d). The slow progression of the initial infarction at the territory of posterior inferior cerebellar artery might may be a result of small arterial occlusion due to endothelial proliferation (Fig. 1, Fig. 4d, e), and this condition may have partly contributed to the edema. However, massive edema could not be explained by arterial occlusion alone. Together with the fact that the main venous sinuses were patent (Fig. 2) and that venules were widely congested, the massive cerebellar edema may be predominantly due to occlusion of small veins at the subarachnoid space rather than due to occlusion of large veins such as sinuses [10]. The findings on autopsy indicated that the unique progression of the ischemic and edematous lesion on MRI reflected the invasion of the fungi and aggressive fibrosis.

Some cases of CM are known to present with fulminant intracranial pressure elevation, which leads to brain herniation and death [11–16]. Zhu reported that cerebral herniation was observed in 30 (19.5%) of 154 non-HIV patients with CM reviewed in their study. In 30 patients who presented with cerebral herniation, 21 died within a year of the diagnosis and 11 within a week, 10 of whom died due to cerebral herniation. They also found that cerebral herniation was one of the crucial and independent factors for poor prognosis in patients with CM [11]. Thus, although cerebral herniation is known to be the most common cause of death in patients with CM, the mechanisms of herniation are not well described. Hydrocephalus due to impaired absorption or obstruction of CSF may be one of the primary reasons for cerebral herniation [12, 17]. The temporal change of the MRI and the findings from autopsy indicate that subarachnoid vein occlusion is a potential mechanism for massive brain edema and an aggressive clinical course in CM. When the fungi broadly and deeply spread into the sulcus of the cerebellum, inflammatory fibrosis widely occurs and might lead to venule congestion and, ultimately, to life threatening massive brain edema. Infarction in lesions, independent of the vascular territory, may be a sign of the progression of fibrosis of the subarachnoid space and decreased compliance of the brain. To our knowledge, this is the first case demonstrating the clinical course and pathological findings of subarachnoid vein occlusion caused by *C. neoformans.* Further reports are necessary to characterize the time course of *C. neoformans* in the central nervous system.

## Conclusions

In conclusion, we suggest that CM induces subarachnoid small vein occlusion via inflammatory fibrosis, which leads to an aggressive and fatal clinical course. In this case, the temporal change in perifocal edema and ischemic lesions suggested an etiology different from that of a simple arterial infarction. Hence, CM should be considered as a cause of progressive cerebral infarction even in the absence of prior HIV.

## Abbreviations

CM: Cryptococcal meningoencephalitis
DWI: Diffusion weighted image
FLAIR: Fluid-attenuated inversion recovery
GCS: Glasgow coma scale
MRI: Magnetic resonance imaging
